# LEM Domain Containing 1 Acts as a Novel Oncogene and Therapeutic Target for Triple-Negative Breast Cancer

**DOI:** 10.3390/cancers15112924

**Published:** 2023-05-26

**Authors:** Xiangling Li, Shilong Jiang, Ting Jiang, Xinyuan Sun, Yidi Guan, Songqing Fan, Yan Cheng

**Affiliations:** 1 Department of Pharmacy, The Second Xiangya Hospital, Central South University, Changsha 410011, China; 2 Hunan Provincial Engineering Research Centre of Translational Medicine and Innovative Drug, Changsha 410011, China; 3 Department of Pharmacy, Xiangya Hospital, Central South University, Changsha 410008, China; 4 Department of Pathology, The Second Xiangya Hospital, Central South University, Changsha 410011, China; 5 Key Laboratory of Diabetes Immunology, Central South University, Ministry of Education, Changsha 410011, China

**Keywords:** TNBC, LEMD1, ERK, therapeutic target

## Abstract

**Simple Summary:**

Since TNBC shows the worst prognosis and limited treatment options, exploring novel molecular targets is urgently needed for effective treatment of TNBC. In this study, we demonstrated that LEMD1 is highly expressed in TNBC and contributes to poor prognosis of TNBC patients. LEMD1 silencing not only inhibited the proliferation and migration of TNBC cells in vitro, but also abolished tumor formation of TNBC cells in vivo. Mechanistically, LEMD1 promotes the progress of TNBC by activating the ERK signaling pathway. Knockdown of LEMD1 renders TNBC cells more sensitive to paclitaxel. Our results uncovered LEMD1 as a novel oncogene in TNBC, and targeting LEMD1 might be a promising therapeutic approach for the effective treatment of TNBC patients.

**Abstract:**

Breast cancer is the most common deadly malignancy in women worldwide. In particular, triple-negative breast cancer (TNBC) exhibits the worst prognosis among four subtypes of breast cancer due to limited treatment options. Exploring novel therapeutic targets holds promise for developing effective treatments for TNBC. Here, we demonstrated for the first time that LEMD1 (LEM domain containing 1) is highly expressed in TNBC and contributes to reduced survival in TNBC patients, through analysis of both bioinformatic databases and collected patient samples. Furthermore, LEMD1 silencing not only inhibited the proliferation and migration of TNBC cells in vitro, but also abolished tumor formation of TNBC cells in vivo. Knockdown of LEMD1 enhanced the sensitivity of TNBC cells to paclitaxel. Mechanistically, LEMD1 promoted the progress of TNBC by activating the ERK signaling pathway. In summary, our study revealed that LEMD1 may act as a novel oncogene in TNBC, and targeting LEMD1 may be exploited as a promising therapeutic approach to enhance the efficacy of chemotherapy against TNBC.

## 1. Introduction

Breast cancer is the most frequent female malignancy around the world, with high incidence and fatality [1]. Estrogen receptor (ER), progesterone receptor (PR) and epidermal growth factor receptor (Her2) on tumor cell surfaces are the main targets for therapeutic treatment of breast cancer. However, with the expression deficiency of these three key markers, triple negative breast cancer (TNBC) patients can benefit from neither endocrine therapy nor HER2-targeted therapy [2]. Chemotherapy still remains the mainstay systemic treatment for TNBC patients, despite its poor efficacy and severe toxic effect [3,4]. Moreover, TNBC patients share the clinical features of high invasiveness and metastatic potential, which also plagues the effectiveness of current treatment regimens [4]. Therefore, discovering effective targeted therapies for TNBC treatment is urgently needed.

LEM domain containing 1 (LEMD1) belongs to the cancer-testis antigen (CTA) family [5]. Since LEMD1 was first isolated from colorectal cancer in 2004, the role it plays during tumor progression has attracted widespread concern among researchers. It has been reported that LEMD1 is aberrantly overexpressed in a series of malignancies and is correlated with worse prognosis of tumor patients such as prostate cancer, colorectal cancer, gastric cancer and pancreatic cancer [5,6,7,8,9]. LEMD1 exerts oncogenic effects during tumorigenesis of various cancers. For example, the elevated expression of LEMD1 contributes to cell proliferation in gastric cancer [7]. LEMD1 also facilitates invasion and epithelial–mesenchymal transition (EMT) in oral squamous cell carcinoma, thyroid cancer and pancreatic cancer [8,10,11]. Transcriptome data has revealed that LEMD1 is highly expressed in cancer stem cells (CSCs)/cancer-initiating cells (CICs) and is important for the maintenance of CSCs/CICs in colorectal cancer [12]. Taken together, these studies demonstrate that LEMD1 plays a pivotal role in promoting tumor progression, suggesting its clinical value as a potential prognostic biomarker and therapeutic target in diverse tumors. Nevertheless, the biological function of LEMD1 in breast cancer has not been elucidated yet.

In this study, we innovatively explored the expression and the prognostic value of LEMD1 in TNBC. In addition, we elucidated the biological role and the clinical value of LEMD1 in the progress of TNBC, and also performed further analysis to clarify the underlying mechanism. In conclusion, our results revealed the biological function of LEMD1 during tumorigenesis of TNBC and provided a potential therapeutic strategy of targeting LEMD1 for TNBC treatment.

## 2. Materials and Methods

### 2.1. Bioinformatics Analysis

A series of genomic datasets in breast cancer, including GSE65216, GSE20713, GSE45827 and GSE76275, downloaded from Gene Expression Omnibus (GEO), provide gene expression profiles of tumor samples. In detail, GEPIA 2 (Gene Expression Profiling Interactive Analysis 2) was used to compare gene expressions between tumor and normal tissues of breast cancer samples, and to analyze the influence of LEMD1 expression on the survival outcome in multiple cancers. The association of LEMD1 and the overall survival of TNBC patients was completed by Kaplan–Meier Plotter. DNMIVD [13] was used to analyze LEMD1 methylation levels and its relationship with patients’ survival. TIMER [14] is a web server providing data on LEMD1 expression in tumor tissues and corresponding normal tissues among various cancers. Additionally, we explored the influence of gene set expression on anti-cancer drug resistance by GSCALite server [15]. ROC plotter server [16] was utilized to analyze the correlation between the expression of LEMD1 and the therapeutic responses of TNBC patients to chemotherapy.

### 2.2. Cell Lines and Culture

The human breast cancer cell lines MDA-MB-468, BT549 and HCC1806 were cultured in RPMI-1640 medium. HEK-293T and MDA-MB-231 were cultured in DMEM medium. MDA-MB-436 was cultured in L-15 medium. These cells were all cultured in media supplemented with 10% fetal bovine serum (Gibco), penicillin (100 U/mL) and streptomycin (100 µg/mL). Additionally, all cell lines were maintained at 37 °C in a humidified atmosphere of 5% CO_2_/95% air and identified via the STR method.

### 2.3. siRNA, shRNA, CRISPR/Cas9 Lentivirus and Plasmid Transfection

RiboBio (Guangzhou, China) supplied the siRNA targeting LEMD1. Cells in good growth condition were plated in 6-well plates and then transfected with siRNA according to the manufacturer’s protocol. For the construction of stable silencing cells, the LEMD1 shRNA plasmids were transfected into HEK-293T cells and the supernatant of HEK-293T cells was added into MDA-MB-231 cells, following by screening with 2 μg/mL of puromycin for a week. For the construction of LEMD1-KO cells, the CRISPER/CAS9 lentiviruses were transfected into MDA-MB-468 cells for 72 h, and then selected with 8 μg/mL of puromycin for a week. The plasmids involved in this study were transfected by Lipofectamine 8000 (Beyotime, Shanghai, China) reagent.

### 2.4. Western Blot

Cells were lysed with RIPA buffer containing protease inhibitor and phosphatase inhibitor at 4 °C for 30 min, and then centrifuged at 12,000× *g* for 15 min at 4 °C. Proteins (20 µg) were run on SDS-PAGE gels and then transferred to PVDF membranes. After blocking with skim milk, the PVDF membranes were incubated in 5% BSA at 4 °C overnight with the corresponding primary antibodies as follows: LEMD1 (GTX16303, 1:1000, Genetex, Irvine, CA, USA), LEMD1 (ab201206, 1:1000, Abcam, Cambridge, UK), Lamin B1 (sc-377000, 1:500, Santa Cruz, Santa Cruz, CA, USA), E-cadherin (ET1607-75, 1:1000, Huabio, Hangzhou, China), N-cadherin (ET1607-37, 1:2000, Huabio, Hangzhou, China), vimentin (M1412-1, 1:1000, Huabio, Hangzhou, China), Bax (50599-2-lg, 1:1000, Proteintech, Chicago, IL, USA), Bcl-2 (#15071S, 1:1000, CST, Boston, MA, USA), ERK (#0102S, 1:1000, CST), p-ERK (#9101S, 1:1000, CST), Flag (M185-3L, 1:1000, MBL, Beijing, China), β-actin (20536, 1:7000, Proteintech), GAPDH (GB11002, 1:1000, Servicebio, Wuhan, China), following by treatment with the corresponding secondary antibody at room temperature for 1 h. Finally, the protein signals were visualized with ECL reagent.

### 2.5. RNA Isolation, Reverse Transcription (RT) and Real-Time PCR

Total RNA from cell lines was isolated using Trizol reagent (Invitrogen, Carlsbad, CA, USA). A total of 1 μg of RNA was reverse-transcribed using the PrimeScript RT Reagent Kit (Takara, Dalian, China). Real-time PCR was carried out using SYBR Green (Takara). The mRNA expression of all target genes was normalized to GAPDH.

### 2.6. Cell Viability Assays

The cells were plated in 96-well plates and treated with paclitaxel for 72 h. Then, cell viability was measured with CCK8 (Bimake, Shanghai, China) reagent. The cells were incubated at 37 °C for 2 h after adding CCK8. Additionally, the OD value at 450 nm was detected to determine cell viability.

### 2.7. Clonogenic Assay

Cells were plated in 6-well plates (500 cells/well) and treated with paclitaxel for 24 h. After the incubation of about 15 days, 4% paraformaldehyde and crystal violet were used to fix and stain the cell colonies.

### 2.8. 5-Ethynyl-2′-deoxyuridine Assay

According to the instruction, the cells were incubated with 5-Ethynyl-2′-deoxyuridine assay (EDU, RiboBio, Chengdu, China) for 2 h at 37 °C and fixed with 4% paraformaldehyde for 30 min, followed by permeating with 0.5% Triton X-100 for 10 min at room temperature. Then, cells were stained with 1× Apollo dye reaction solution for 30 min and treated with Hoechst for 30 min away from light at room temperature. Finally, a fluorescent microscope was needed to capture the images.

### 2.9. Wound Healing Assay

To measure cell migration, cells were plated in 6-well plates and maintained at 37 °C. A line was drawn with sterile 10 μL tips on the monolayer cells and rinsed with medium to remove any floating cell debris when the cells reached 100% confluence. Images were captured at the indicated time. The wound healing rate (%) was calculated and analyzed.

### 2.10. Cell Migration and Invasion

The cells’ migration and invasion ability were examined using Matrigel and Transwell plates. The cells (3 × 10^4^ cells/well) were inoculated into the upper chamber of the Transwell plate in serum-free medium. Particularly, the upper chambers were coated with Matrigel (BD, Biosciences, Chongqing, China) for the invasion assay. The medium containing 20% FBS was added to the lower chamber. After incubation for 24 h (migration) and 72 h (invasion), the cells on the submembrane surface were fixed with 4% paraformaldehyde and stained with crystal violet. The extra cells and the Matrigel were removed with cotton swabs, and then the invaded and migrated cells were photographed and counted.

### 2.11. Immunofluorescence Staining

MDA-MB-468 cells were seeded on a glass coverslip and fixed in 4% paraformaldehyde for 25 min at room temperature. Next, the cells were blocked in 5% bovine serum albumin (BSA) for 2 h and incubated with anti-LEMD1 antibody (GTX16303, 1:200, Genetex) at 4 °C overnight, followed by Alexa Fluor 594 dye-conjugated anti-rabbit IgG antibody. At the end of incubation, the cell nuclei were stained with DAPI for 2 min. The coverslip was washed with phosphate-buffered saline (PBS) and images were detected and captured using a confocal microscope.

### 2.12. Tissue Microarray (TMA) and Immunohistochemistry (IHC)

The Second Xiangya Hospital of Central South University (Changsha, China) provided the clinical specimens involved in this study. Additionally, all patients were given informed consent before the experiment. For TMA, samples embedded with paraffin were arranged by a tissue-arraying instrument; each sample was arranged in three 1-mmdiameter cores to reduce tissue loss and minimize tumor heterogeneity. IHC staining for LEMD1 (Abcam, ab201206, 1:100) was conducted by using the DAKO LSAB + System-HRP kit (DAKO, Copenhagen, Denmark) according to the protocols. The protein expression of LEMD1 was detected with a polyclonal antibody at a dilution of 1:200. IHC scores were evaluated by two independent pathologists in a blinded situation.

### 2.13. Semi-Quantitative Analysis of TMA and IHC Staining

Two independent pathologists, who were experienced in assessing IHC and were blinded to the clinical outcome of the involved patients, were invited to evaluate all of the samples. We determined the expression of LEMD1 by using two indicators: the intensity and the range of the staining. The percentage of immunoreactive cells was rated as follows: 0 points, <10% positive cells; 1 point, 10–40% positive cells; 2 points, 40–70% positive cells; 3 points, >70% positive cells. The staining intensity was rated as follows: 0 (no staining), 1 (weak staining), 2 (moderate staining), 3 (strong staining). The two scores were multiplied and used to divide the patients into a high expression group (score ≥ 3) and a low expression group (score < 3) of LEMD1. The final score is the mean of the scores assigned by the two pathologists [17,18].

### 2.14. RNA-Sequencing and Signaling Pathway Assays

Total RNA was extracted from MDA-MB-468 cell lines treated with LEMD1 siRNA or a negative control. After confirming the quality, purity and integrity of the RNA, a cDNA library was established from ~1 μg of total RNA. Then, the library was sequenced on an Illumina Novaseq6000 using 2 × 150 bp paired-end sequencing chemistry. The Kyoto Encyclopedia of Genes and Genomes (KEGG) pathway enrichment analysis was performed by analyzing the obtained genes. All services were provided by LC Biotech Corporation (Hangzhou, China).

### 2.15. Animal Studies

MDA-MB-231 cells with stable LEMD1 knockdown and control MDA-MB-231 cells were administered subcutaneously into 5-week-old female BALB/c nude mice (1 × 10^6^ cells in 100 μL medium). Tumor sizes and body weights were measured every other day. Tumor volume was calculated as length × width^2^ × (π/6). The subcutaneous tumors were excised, weighed and captured at the termination of the experiment.

### 2.16. Statistical Analysis

We analyzed the data using SPSS 22.0 and GraphPad Prism 8.0 software. Pearson’s χ^2^ test was used to analyze the association between LEMD1 expression and the clinicopathological features of breast cancer patients. A two-tailed unpaired Student *t* test was conducted to analyze the differences between two groups. *p* < 0.05 was considered statistically significant.

## 3. Results

### 3.1. The High Expression of LEMD1 Is Associated with Poor Prognosis in TNBC

**Figure 1:**
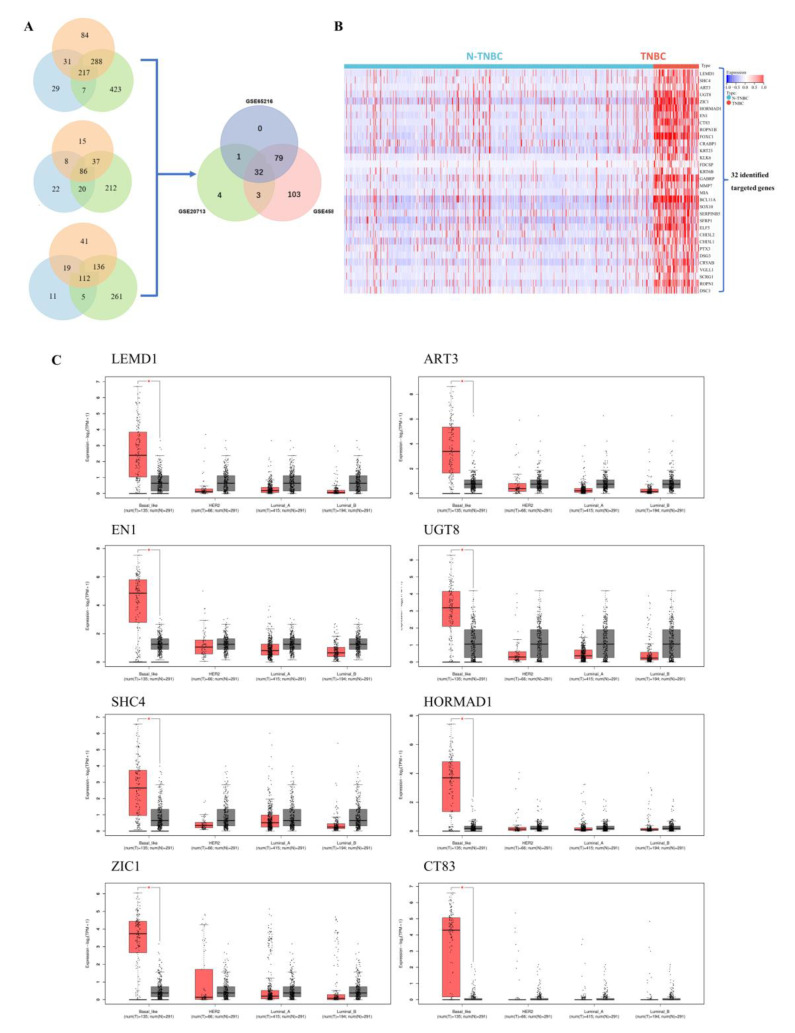
Screening of the high expression genes in TNBC. (**A**) Venn diagram of the differential genes between TNBC and non-TNBC samples in GEO datasets. (**B**) The expressions of 32 genes in TNBC and non-TNBC in the TCGA-BRCA cohort. Heat maps represent gene expression levels. (**C**) The GEPIA analysis of 8 genes with specific high expression in TNBC. Red represents tumor tissues and black represents corresponding normal tissues. * *p* < 0.05.

In order to explore the genes involved in the progress of TNBC, we first screened 32 genes with specific high expressions in TNBC compared with other subtypes of breast cancer by analyzing GEO datasets including GSE65216, GSE20713 and GSE45827 (log_2_FC > 1.5) (Figure 1A). The high expressions of these 32 genes in TNBC were also verified in the Cancer Genome Atlas Breast Invasive Carcinoma (TCGA-BRCA) (Figure 1B). We further analyzed the expressions of 32 genes in TNBC and normal breast tissue, and found that 8 genes (*LEMD1*, *ART3*, *EN1*, *UGT8*, *SHC4*, *HORMAD1*, *ZIC1*, *CT83*) showed higher expression in TNBC tissue compared to the normal breast tissue by GEPIA analysis (Figure 1C and Supplementary Figure S1). Considering the role of LEMD1 in breast cancer remains unknown, we further explored the biologic function and the clinical value of LEMD1 in TNBC.

**Figure 2:**
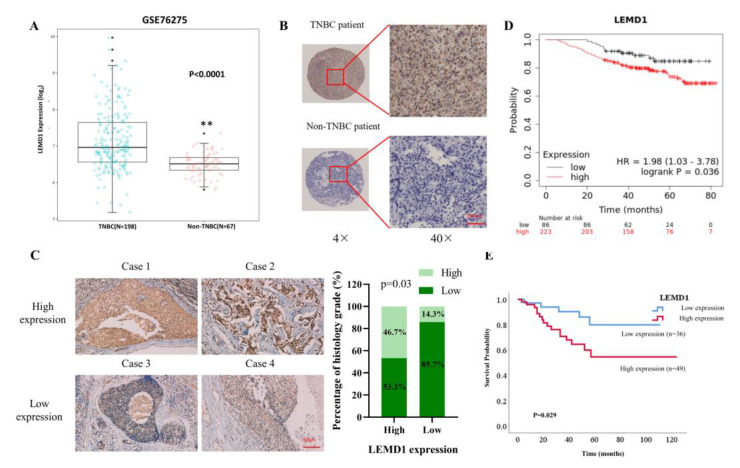
The relationship between LEMD1 and clinicopathological features of TNBC patients. (**A**) LEMD1 expressions of TNBC and non-TNBC patients in GSE76275 from GEO database. ** *p* < 0.01. (**B**) Representative images of LEMD1 from a tissue microarray containing 80 breast cancer patients. (**C**) Representative IHC images of LEMD1 in 63 clinical samples of TNBC patients. Scale bar, 50 μm. The positive correlation between LEMD1 expressions and the histology grade in TNBC patients. (**D**) Kaplan–Meier plot of the overall survival (OS) of TNBC patients from TCGA database. (**E**) Kaplan–Meier plots of the OS from a tissue microarray containing 85 breast cancer patients.

Next, we confirmed LEMD1 expression in breast cancer tissues. Firstly, data from an independent breast cancer dataset consistently shows that LEMD1 is significantly upregulated in TNBC tumors compared to non-TNBC tumors (Figure 2A). Moreover, higher protein expressions of LEMD1 were observed in TNBC tissues compared to non-TNBC tissues in a tissue microarray (TMA) containing 80 breast cancer specimens we collected from the Second Xiangya Hospital (Figure 2B). In addition, we examined the protein expressions of LEMD1 in 63 TNBC patients from the Second Xiangya Hospital by immunohistochemistry (IHC) staining, and further analyzed the relationship between LEMD1 expressions and the clinicopathological features of TNBC patients. As shown in Figure 2C and Table 1, we found that high expression of LEMD1 was markedly associated with higher histology grade of TNBC patients, suggesting that LEMD1 may act as a cancer-promoting factor in TNBC. The survival curve, analyzed by Kaplan–Meier Plotter database, revealed that TNBC patients with higher LEMD1 expression showed worse overall survival (Figure 2D). In addition, the low expression of LEMD1 was positively correlated with improved survival in breast cancer patients, as shown by analysis of the clinical data of 85 patients we collected (Figure 2E). Since LEMD1 is one of the family members of cancer testis antigen that is often regulated by DNA demethylation, as reported [19,20,21], we analyzed the methylation level of LEMD1 in breast cancer. As shown in Supplementary Figure S2A,B, LEMD1 is hypomethylated in breast cancer tissues compared with normal tissues, and its hypomethylation was positively correlated with reduced disease-free interval (DFI) in breast cancer patients. These results indicated that LEMD1 is overexpressed and acts as a poor prognostic factor in TNBC.

### 3.2. Pan-Cancer Analysis of LEMD1 Expression and Prognosis

In view of the critical role of LEMD1 in TNBC, we performed pan-cancer analysis to gain a more comprehensive understanding of LEMD1 in various cancers. Firstly, we analyzed LEMD1 expression in various cancers by comparing the transcriptome data between tumor tissues and corresponding adjacent normal tissues. The result shows that LEMD1 was highly expressed in various cancers, including bladder urothelial carcinoma (BLCA), invasive breast carcinoma (BRCA), cholangiocarcinoma (CHOL), colon adenocarcinoma (COAD), esophageal carcinoma (ESCA), glioblastoma multiforme (GBM), head and neck squamous cell carcinoma (HNSC), kidney chromophobe (KICH), kidney renal clear cell carcinoma (KIRC), kidney renal papillary cell carcinoma (KIRP), lung adenocarcinoma (LUAD), lung squamous cell carcinoma (LUSC), pancreatic adenocarcinoma (PAAD), prostate adenocarcinoma (PRAD), rectal adenocarcinoma (READ), stomach adenocarcinoma (STAD), thyroid carcinoma (THCA) and uterine corpus endometrial carcinoma (UCEC) (Supplementary Figure S3A), suggesting that LEMD1 may serve as a potential key regulator during tumorigenesis. Next, we analyzed the prognostic value of LEMD1 in pan-cancer and found that high expression of LEMD1 was significantly associated with worse OS rate in COAD (*p* = 0.047), KIRC (*p* = 0.0065), KIRP (*p* = 0.023) and PAAD (*p* = 0.0027) (Supplementary Figure S3B). These results revealed that LEMD1 is upregulated and may serve as a poor prognostic factor in multiple cancers.

### 3.3. LEMD1 Promotes the Progression of TNBC In Vitro and In Vivo

**Figure 3:**
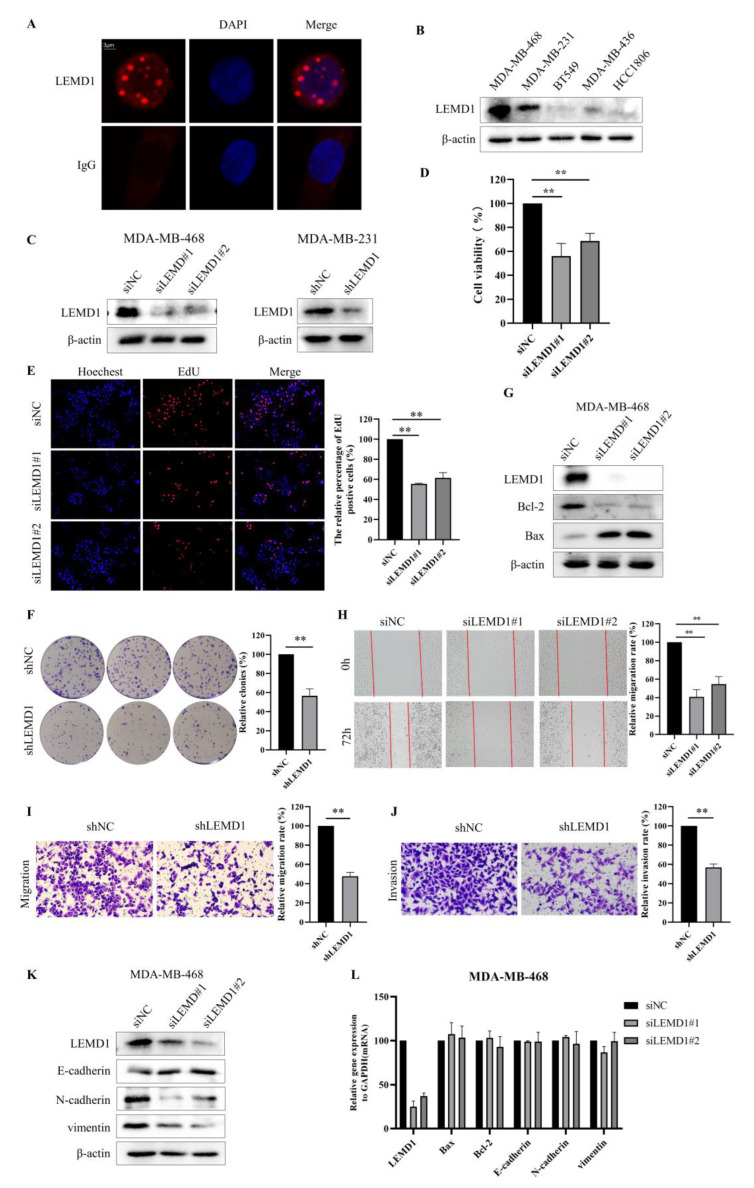
LEMD1 promotes the proliferation and migration of TNBC cells. (**A**) The subcellular location of LEMD1 was examined in MDA-MB-468 cells by immunofluorescence staining. IgG antibody was used for negative control. The location of the nucleus was indicated by DAPI. (**B**) The expressions of LEMD1 in TNBC cell lines were examined by Western blot, β-actin was used as a loading control. (**C**) The expressions of LEMD1 in MDA-MB-468 cells were knocked down by siRNA, and the expressions of LEMD1 in MDA-MB-231 cells were knocked down by shRNA. (**D**) CCK-8 reagent was applied to examine cell viability of MDA-MB-468 cells. ** *p* < 0.01, n = 3. (**E**) Cell proliferation of MDA-MB-468 cells was measured using EdU. Magnification ×100. ** *p* < 0.01, n = 3. (**F**) The colony formation assay in LEMD1-knockdown MDA-MB-231 and the corresponding negative control cells were used to examine cell proliferation. ** *p* < 0.01, n = 3. (**G**) Western blot analysis of the expressions of Bcl-2 and Bax in MDA-MB-468 cells, β-actin was used as a loading control. (**H**) Wound healing assays of MDA-MB-468 cells. Magnification ×100. ** *p* < 0.01, n = 3. Transwell migration assays (**I**) and Transwell invasion assays (**J**) in LEMD1-konckdown MDA-MB-231 cells and their corresponding negative control cells. Magnification ×200. ** *p* < 0.01, n = 3. (**K**) Western blot analysis of the expressions of E-cadherin, N-cadherin and vimentin in MDA-MB-468 cells, β-actin was used as a loading control. (**L**) qPCR analysis of the mRNA expressions of Bax, Bcl-2, E-cadherin, N-cadherin and vimentin in MDA-MB-468 cells. The original western blot figures could be found in Supplementary Figure S6A.

We next sought to investigate the biological functions of LEMD1 in TNBC. Firstly, we examined the subcellular location of LEMD1, and found that LEMD1 is located in the nucleus via immunofluorescence staining (Figure 3A). We further detected LEMD1 expressions in several TNBC cell lines and found that LEMD1 was highly expressed in MDA-MB-468 and MDA-MB-231 cells (Figure 3B). Therefore, we used siRNA/shRNA to knockdown LEMD1 expression in MDA-MB-468 cells and MDA-MB-231 cells, respectively (Figure 3C). Western blot analysis of LEMD1-knockdown cells also verified the nuclear localization of LEMD1 in TNBC cells (Supplementary Figure S4A). Importantly, we found that knockdown of LEMD1 significantly inhibited the cell viability in MDA-MB-468 and MDA-MB-231 cells (Figure 3D and Supplementary Figure S4B). The numbers of cells positive for EdU staining were also significantly decreased in TNBC cells with LEMD1 knockdown (Figure 3E and Supplementary Figure S4C). Furthermore, the inhibited proliferation induced by LEMD1 knockdown was also confirmed via a colony formation assay examining long-term survival (Figure 3F). These results indicated that LEMD1 silencing inhibited the proliferation of TNBC cells. We further explored whether LEMD1 could regulate the apoptosis of TNBC cells. As shown in Figure 3G and Supplementary Figure S4D, LEMD1 silencing increased cell apoptosis, as evidenced by the increased expression of Bax and the decreased expression of Bcl-2.

Moreover, we investigated the effect of LEMD1 knockdown on cell migration and invasion. Figure 3H and Supplementary Figure S4E,F show that the migration rates of TNBC cells in LEMD1-knockdown or knockout group were significantly decreased compared to those in control group. Transwell experiments demonstrated that migration and invasion were inhibited in TNBC cells with LEMD1 silencing (Figure 3I,J). We then tested the impact of LEMD1 knockdown on the epithelial–mesenchymal transition (EMT) process of TNBC cells. We found that LEMD1 silencing increased the expression of E-cadherin and decrease the expressions of N-cadherin and vimentin (Figure 3K and Supplementary Figure S4G), suggesting that LEMD1 promotes the EMT process of TNBC cells. Furthermore, we found that there is no significant change in the mRNA levels of Bax, Bcl-2, E-cadherin, N-cadherin and vimentin in cells with LEMD1 knockdown (Figure 3L and Supplementary Figure S4H), indicating that the regulation of LEMD1 on the expressions of the above proteins is a post-transcriptional modification. Taken together, these results demonstrated that LEMD1 functions as a promotive factor in the proliferation, migration and invasion of TNBC cells.

**Figure 4:**
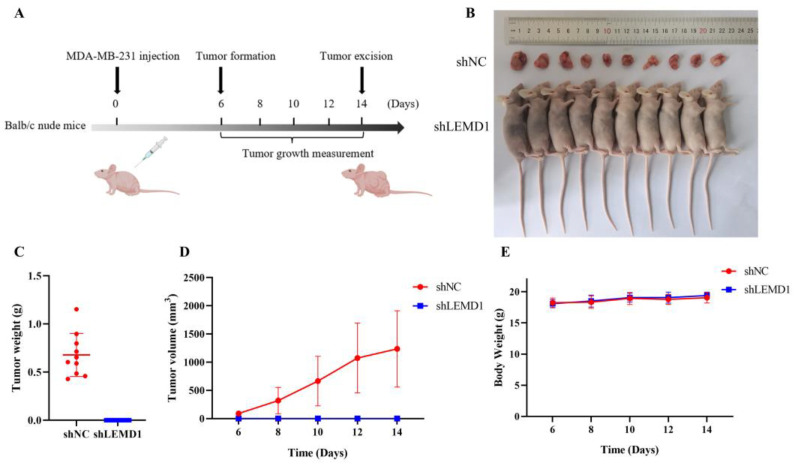
LEMD1 promotes tumor growth in vivo. shNC and shLEMD1 transfected MDA-MB-231 cells were administered subcutaneously into 5-week-old female Balb/c nude mice. (**A**) Diagram of the in vivo experiment. (**B**–**D**) The tumor sizes were monitored on the indicated days. After 2 weeks, the subcutaneous tumors were excised, weighed and photographed. (**E**) The body weight of the mice was recorded during the experiment.

To extend the in vitro observations, we constructed a subcutaneous xenograft model in female nude mice to investigate the influence of LEMD1 silencing on tumor growth in vivo (Figure 4A). As shown in Figure 4B–D, no tumor formation was observed in MDA-MB-231 cells with knockdown of LEMD1 expression, indicating that LEMD1 silencing totally abolished tumor formation in vivo. There was no significant change in body weight in the mice of the two groups (Figure 4E). Collectively, these results confirmed that LEMD1 promotes the progression of TNBC.

### 3.4. LEMD1 Promotes the Cell Proliferation and Invasion by Activating ERK Signaling Pathway in TNBC

**Figure 5:**
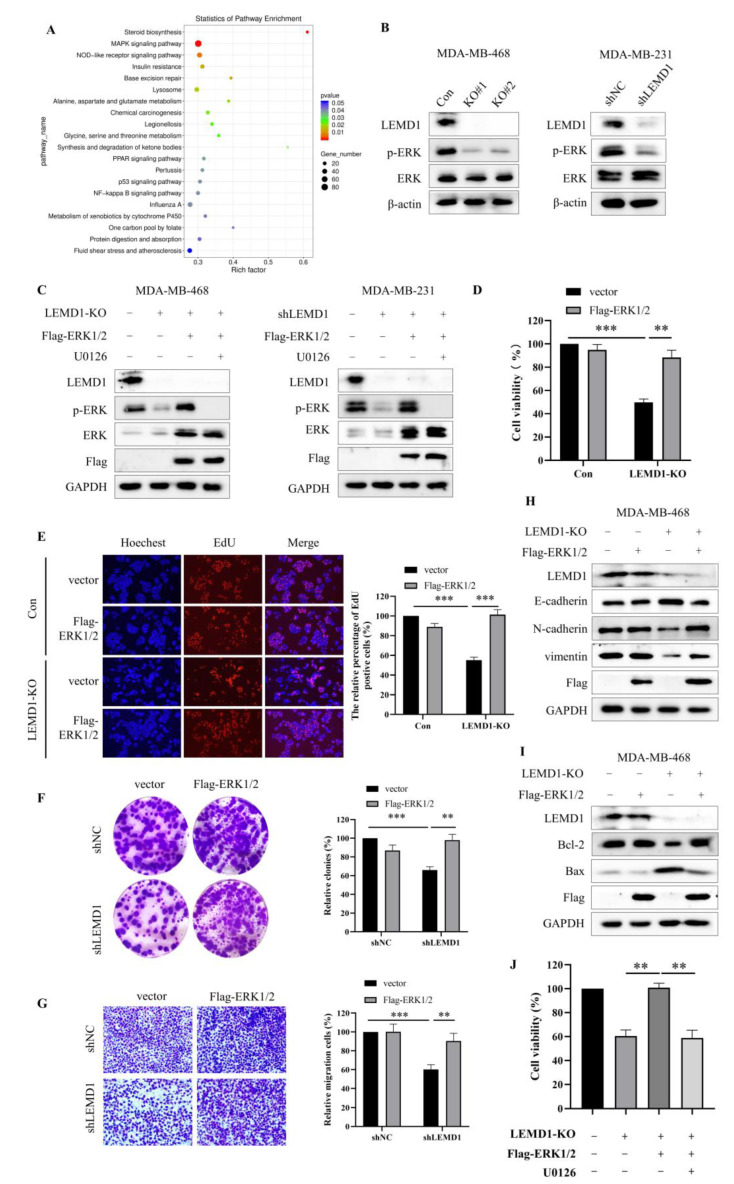
LEMD1 promotes cell proliferation and invasion by activating ERK in TNBC. (**A**) Signaling pathway enrichment analysis of siNC and siLEMD1 MDA-MB-468 cells by RNA-seq. (**B**) LEMD1 was knocked out by LEMD1-targeting CRISPER/CAS9 system in MDA-MB-468 cells and was knocked down by LEMD1 shRNA in MDA-MB-231 cells. The expressions of ERK, p-ERK were examined by Western blot. β-actin was used as a loading control. (**C**) LEMD1-knockout MDA-MB-468 cells and LEMD1-knockdown MDA-MB-231 cells were transfected with ERK plasmid and then treated with U0126, an ERK inhibitor. The expressions of ERK, p-ERK, Flag were examined by Western blot. GAPDH was used as a loading control. LEMD1-knockout MDA-MB-468 cells were transfected with ERK plasmid or empty vector plasmid. Cell viability was measured using the CCK8 assay (**D**) and EdU assay (**E**), magnification ×200. Additionally, LEMD1-knockdown MDA-MB-231 cells were transfected with ERK plasmid or empty vector plasmid. Cell proliferation was measured via colony formation assay (**F**). Cell migration ability was measured via Transwell migration assay (**G**). Magnification ×200. (**H**) The protein expressions of EMT markers including E-cadherin, N-cadherin, vimentin, and (**I**) The expressions of Bcl-2 and Bax in LEMD1-knockout MDA-MB-468 cells were measured via Western blot. GAPDH was used as a loading control. (**J**) LEMD1-knockout MDA-MB-468 cells were transfected with ERK plasmid and then treated with U0126 for 24 h. Cell viability was measured using the CCK8 assay. ** *p* < 0.01, *** *p* < 0.001, n = 3. The original western blot figures could be found in Supplementary Figure S6B.

To explore the mechanism underlying the promotive effect of LEMD1 on the progression of TNBC, RNA-seq was conducted to detect the differentially expressed genes in LEMD1-silenced and control MDA-MB-468 cells. MAPK signaling pathway, an important oncogenic pathway regulating ubiquitous signal transduction process in cancers [22], showed significant differential expression in KEGG pathway enrichment analysis (Figure 5A). MAPK consists of ERK, p38, JNK and ERK5. Furthermore, protein–protein network analysis showed that there are close associations between ERK1/2 and the members of MAPK signaling pathway detected by RNA-seq (Supplementary Figure S5A), suggesting that ERK may mediate the carcinogenic role of LEMD1 in TNBC. To validate this hypothesis, we detected the protein expressions of ERK and p-ERK in LEMD1-knockout (KO) and LEMD1-knockdown (KD) cells. Figure 5B showed that LEMD1 knockout or knockdown remarkably down-regulated the expression of p-ERK, but did not change the expression of ERK, indicating that LEMD1 can activate ERK. Next, LEMD1-knockout and LEMD1-knockdown cells were transfected with ERK plasmid and then treated with the ERK inhibitor, U0126. Figure 5C showed that overexpression of ERK increased the expressions of ERK and p-ERK, and the increased p-ERK induced by ERK overexpression can be reversed by U0126. To investigate whether ERK is involved in the regulation of LEMD1 on tumor progression, cell proliferation and migration were measured. As shown in Figure 5D–G and Supplementary Figure S5B,C, the inhibitory effects on the cell proliferation and migration induced by LEMD1-KD/KO were reversed by ERK overexpression in TNBC cells. It has been reported that ERK activation can promote EMT process in various cancers [23,24,25]. ERK activity can also inhibit cancer cell apoptosis by increasing Bcl-2 expression and decreasing Bax expression [26,27]. Consistently, we found that the regulation of E-cadherin, N-cadherin and vimentin expressions by LEMD1-KO/KD was significantly abolished by overexpression of ERK (Figure 5H and Supplementary Figure S5D). Moreover, the increase in Bax expression and the decrease in Bcl-2 expression induced by LEMD1-KO/KD were also rescued by ERK overexpression (Figure 5I and Supplementary Figure S5E). Moreover, cell viability assay also showed that the rescue effect of ERK overexpression in LEMD1-KO/KD cells could be canceled by U0126 treatment (Figure 5J and Supplementary Figure S5F), further confirming the crucial role of ERK activation in mediating the oncogenic role of LEMD1 in TNBC cells. Taken together, these data suggested that LEMD1 promotes the progression of TNBC by activation ERK signaling pathway.

### 3.5. LEMD1 Knockdown Enhances the Chemosensitivity of TNBC Cells to Paclitaxel

**Figure 6:**
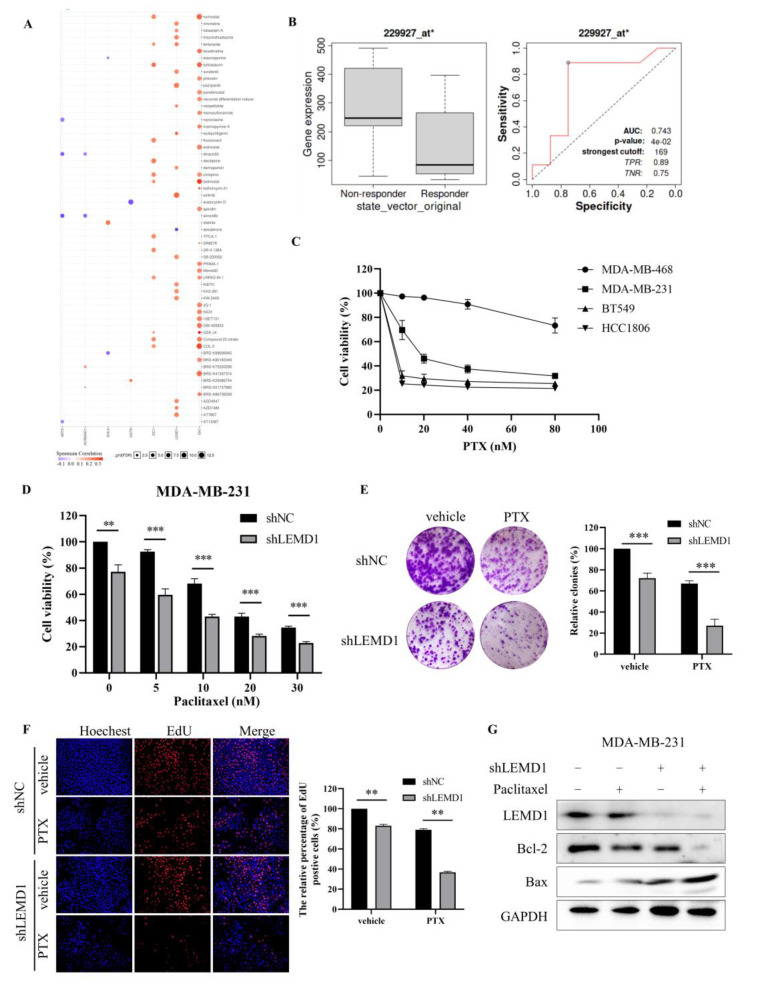
Knockdown of LEMD1 enhances the sensitivity of TNBC cells to paclitaxel. (**A**) Drug resistance analysis of LEMD1, ART3, UGT8, SHC4, HORMAD1, EN1, ZIC1 and CT83 in breast cancer patients by the GSCALite online tool. Red indicates positive relationship, while purple negative. (**B**) The receiver operating characteristic (ROC) curve between LEMD1 expression and therapeutic responses to chemotherapy in TNBC cohorts, * *p* = 0.04. (**C**) MDA-MB-468, MDA-MB-231, BT549 and HCC1806 cells were treated by paclitaxel with indicated concentration for 72 h. Cell viability was determined via CCK8 assay. (**D**) MDA-MB-231 cells were transfected with LEMD1 shRNA or a negative control, followed by treatment with paclitaxel for 72 h. Cell viability was determined via CCK8 assay. ** *p* < 0.01, *** *p* < 0.001, n = 3. (**E**) MDA-MB-231 cells were transfected with LEMD1 shRNA and treated with 10 nM paclitaxel. The colony formation assay was conducted. *** *p* < 0.001, n = 3. (**F**) MDA-MB-231 cells were transfected with LEMD1 shRNA or a negative control and were treated with 10 nM paclitaxel for 72 h. Then, the cells were subjected to an EdU assay. Magnification ×200. ** *p* < 0.01, n = 3. (**G**) MDA-MB-231 cells were transfected with LEMD1 shRNA and were treated with 10 nM paclitaxel for 72 h, the levels of Bcl-2 and Bax were analyzed by Western blot. GAPDH was used as a loading control. The original western blot figures could be found in Supplementary Figure S6C.

In the previous screening process of the target gene, we found that the high expression of LEMD1 was associated with anti-cancer drug resistance in breast cancer cell lines via genomic analysis (Figure 6A), which inspired us to explore the role of LEMD1 in chemotherapy resistance. Considering that chemotherapy remains the major means for TNBC treatment, we further investigated whether LEMD1 is involved in the regulation of the sensitivity of chemotherapeutic drugs in TNBC cells. Firstly, we evaluated the effect of LEMD1 on chemotherapeutic responses of TNBC patients by ROC plotter server. Figure 6B shows that TNBC patients with low LEMD1 expression benefited more from chemotherapies. Next, we further examined the impact of LEMD1 expression on the sensitivity of TNBC cells to paclitaxel, the main chemotherapeutic used for the treatment of TNBC. Figure 6C showed that BT549 and HCC1806 cells with lower LEMD1 expression were more sensitive to paclitaxel than MDA-MB-468 and MDA-MB-231 cells with higher LEMD1 expression, indicating the role of LEMD1 in promoting paclitaxel resistance of TNBC cells. Further experiments showed that LEMD1 knockdown markedly reduced the viability of MDA-MB-231 cells treated with paclitaxel (Figure 6D). The sensitization effect of LEMD1 knockdown on paclitaxel was also confirmed by colony formation assay and EdU assays (Figure 6E,F). LEMD1 knockdown further down-regulated the expression of Bcl-2, increased the expression of Bax compared to paclitaxel alone treatment (Figure 6G). These findings suggested that LEMD1 silencing enhances the efficacy of paclitaxel in TNBC cells.

## 4. Discussion

Due to the clinical characteristics of lack of an appropriate target and rapid progression of patients, the treatment of TNBC still remains chiefly conventional chemotherapy up to now [4]. This dilemma drives researchers to explore novel molecular markers for targeted therapy. In this study, we demonstrated for the first time that LEMD1 is highly expressed in TNBC and is associated with poor prognosis in breast cancer patients. It has been reported that LEMD1 is upregulated in various kinds of malignancies and facilitates cancer progression [5,6,7,8]. Our pan-cancer analysis consistently showed that LEMD1 is overexpressed in various cancers and is associated with shorter survival of tumor patients. Importantly, knockdown of LEMD1 significantly inhibited the proliferation, migration and invasion of TNBC cells. Strikingly, the TNBC cells with LEMD1 knockdown did not form tumors in vivo. These results suggest that LEMD1 may be explored as a novel oncogene and potential therapeutic target for TNBC treatment.

Next, we aimed to figure out the underlying mechanism of LEMD1 promoting the progression of TNBC. We found that LEMD1 activates ERK, and the ERK-mediated signaling pathway is crucial for the regulation of cell proliferation and migration by LEMD1. Accumulating evidences have demonstrated that ERK/MAPK pathway is activated in about 40% of all human cancers, and ERK activation is indispensable for diverse fundamental cell functions including growth, survival and differentiation [22,28,29,30]. Besides, ERK activity also plays a crucial role in promoting the EMT process [23,24,25] and inhibiting apoptosis of tumor cells [26,27]. We found that the regulatory role in EMT protein, Bcl-2 and Bax, induced by LEMD1 knockdown, can be rescued by ERK overexpression, indicating that LEMD1 promotes cell proliferation and migration by activating ERK-mediated regulation of EMT proteins Bcl-2 and Bax. However, the precise mechanism of LEMD1 regulating ERK remains unclear. Dual-specificity phosphatases (DUSPs) can dephosphorylate many key signaling molecules, including MAPKs [31]. It was evidenced that ERK can be dephosphorylated by several DUSPs including DUSP5 [32], DUSP6 [33], DUSP9 [34] and DUSP15 [35]. Our transcriptome data showed that LEMD1 could downregulate the mRNA of DUSP5 and DUSP6, indicating that LEMD1 may increase ERK phosphorylation by downregulating DUSP5 and DUSP6.

In addition, we found that high expression of LEMD1 was significantly correlated with decreased responsiveness to chemotherapeutics in TNBC. LEMD1 knockdown combined with paclitaxel exhibited a stronger inhibitory effect on tumor cell proliferation compared to treatment with paclitaxel alone. These results revealed that high expression of LEMD1 may facilitate chemoresistance of TNBC cells, and targeting LEMD1 may provide an effective approach to increase the sensitivity of TNBC cells to chemotherapy.

In this study, we revealed for the first time that LEMD1 is overexpressed in TNBC through analysis of bioinformatic data and collected patient specimens. Silencing of LEMD1 not only inhibits the proliferation, migration and invasion abilities of TNBC cells in vitro, but also abolishes tumor formation of TNBC cells in vivo. Targeting of LEMD1 enhances the sensitivity of TNBC cells to paclitaxel. Mechanistically, we found that the ERK signaling pathway was involved in the regulation of LEMD1 on the proliferation and migration of TNBC cells. Our results revealed the promotive role of LEMD1 in the progress of TNBC and provided a potential therapeutic strategy of targeting LEMD1 for TNBC treatment.

## 5. Conclusions

In summary, our findings not only provided insight into understanding the mechanism underlying the regulation of cancer cell proliferation and migration by LEMD1, but also laid a solid foundation for the development of a novel oncogene and therapeutic target for TNBC treatment.

## Figures and Tables

**Table 1:** The relationship between clinicopathological characteristics and LEMD1 expressions in 63 TNBC patients.

Characteristic	LEMD1		*p* Value
	Low	High	
Age, years			
<50	7	27	0.516
≥50	8	21	
Tumor size, cm			
<3	5	27	0.121
≥3	10	21	
Histology Grade			
Low	12	24	0.030
High	2	21	
Lymph node metastasis			
No	7	26	0.612
Yes	8	22	
TNM stage			
Ⅰ/Ⅱ	7	26	0.612
Ⅲ/IV	8	22	

## Data Availability

The datasets presented in this study can be found in online repositories. The names of the repository/repositories and accession number(s) can be found in the article.

## Supplementary Materials

The following supporting information can be downloaded at: https://www.mdpi.com/article/10.3390/cancers15112924/s1, Figure S1: The expressions of other 24 genes in breast cancer from GEPIA; Figure S2: The methylation analysis of LEMD1 in breast cancer. (A) LEMD1 DNA methylation levels in normal and tumor samples of breast cancer patients. (B) The correlation between LEMD1 methylation levels and the disease-free interval (DFI) in breast cancer patients; Figure S3: Pan-cancer analysis of LEMD1 expression and the prognostic value. (A) The mRNA expressions of LEMD1 in cancers from TCGA. (B) GEPIA2 was used to perform the overall survival analysis by LEMD1 expression among various tumors by analyzing data from TCGA database. The survival map and the Kaplan-Meier curves with statistical significance were exhibited; Figure S4: The localization and the oncogenic function of LEMD1 in TNBC cells. (A) The expression of LEMD1 was detected in cytoplasm and nucleus by Western blot. β-actin was used as the cytoplasmic loading control, and Lamin B1 was used as the nuclear loading control. (B) MDA-MB-231 cells were transfected with shRNA or a negative control, CCK-8 reagent was applied to examine cell viability. ** *p* <0.01, n = 3. (C) Cell proliferation of MDA-MB-231 cells was measured using EdU. Magnification, ×100. ** *p* < 0.01, n = 3. (D) Western blot analysis of the expressions of Bcl-2 and Bax in MDA-MB-231 cells, β-actin was used as a loading control. (E) Wound healing assays of LEMD1-knockout MDA-MB-468 cells. Magnification, ×100. ** *p* <0.01, n = 3. (F) Wound healing assays of LEMD1-knockdown MDA-MB-231 cells. Magnification, ×100. ** *p* < 0.01, n = 3. (G) Western blot analysis of the expressions of E-cadherin, N-cadherin and vimentin in MDA-MB-231 cells, β-actin was used as a loading control. (H) qPCR analysis of the mRNA expressions of Bax, Bcl-2, E-cadherin, N-cadherin and vimentin in MDA-MB-231 cells; Figure S5: LEMD1 promotes the cell proliferation and invasion by activating ERK in TNBC. (A) The PPI network for ERK1 (MAPK3), ERK2 (MAPK1) and the 11 differential genes with significant variation in MAPK signaling pathway from RNA-seq. LEMD1-knockdown MDA-MB-231 cells were transfected with ERK plasmid or empty vector plasmid. (B) Cell viability was measured using the CCK8 assay. (C) Cell proliferation was measured by EdU assay. Magnification, ×200. (D) The protein expressions of EMT markers including E-cadherin, N-cadherin, vimentin, and (E) The expressions of Bcl-2 and Bax in LEMD1-knockdown MDA-MB-231 cells were measured by Western blot. GAPDH was used as a loading control. (F) LEMD1-knockdown MDA-MB-231 cells were transfected with ERK plasmid and then treated with U0126 for 24h. Cell viability was measured using the CCK8 assay; Figure S6: The original western blot figures.
