# Supplementary material for: LEM Domain Containing 1 Acts as a Novel Oncogene and Therapeutic Target for Triple-Negative Breast Cancer
Source: Cancers (Basel). 2023 May 26;15(11):2924. doi: 10.3390/cancers15112924 (PMC10251810; doi:10.3390/cancers15112924)
Supplement: Supplementary file 1 [file cancers-15-02924-s001.zip › cancers-2368677-supplementary.pdf]

Supplementary materials:

Title: LEM domain containing 1 acts as a novel oncogene and therapeutic target for triple-negative breast cancer

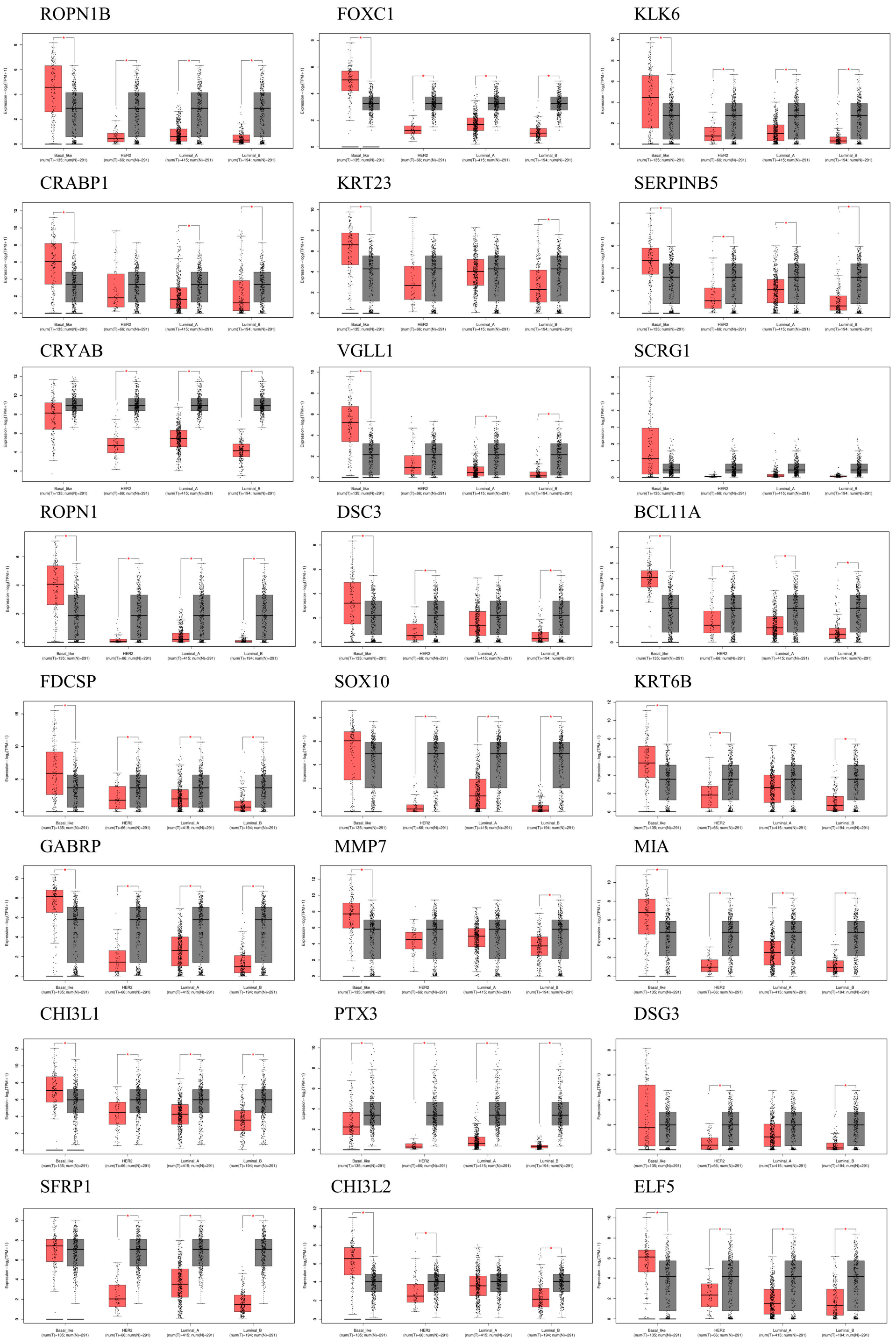

Figure S1. **The expressions of other 24 genes in breast cancer from GEPIA.**

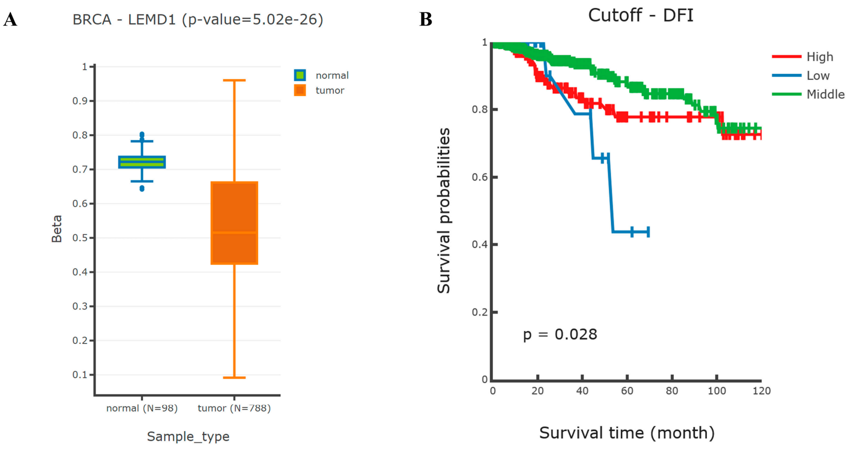

Figure S2. The methylation analysis of LEMD1 in breast cancer. (A) LEMD1 DNA methylation levels in normal and tumor samples of breast cancer patients. **(B)** The correlation between LEMD1 methylation levels and the disease-free interval (DFI) in breast cancer patients.

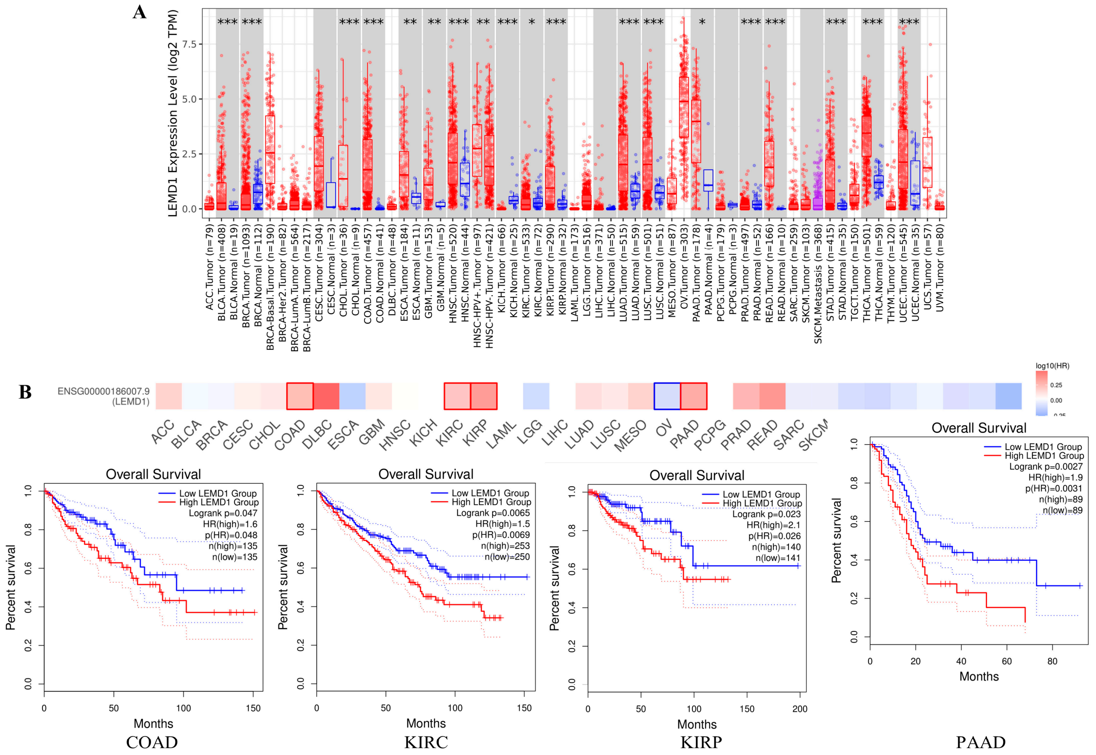

Figure S3. Pan-cancer analysis of LEMD1 expression and the prognostic value. (A) The mRNA expressions of LEMD1 in cancers from TCGA. **(B)** GEPIA2 was used to perform the overall survival analysis by LEMD1 expression among various tumors by analyzing data from TCGA database. The survival map and the Kaplan-Meier curves with statistical significance were exhibited.

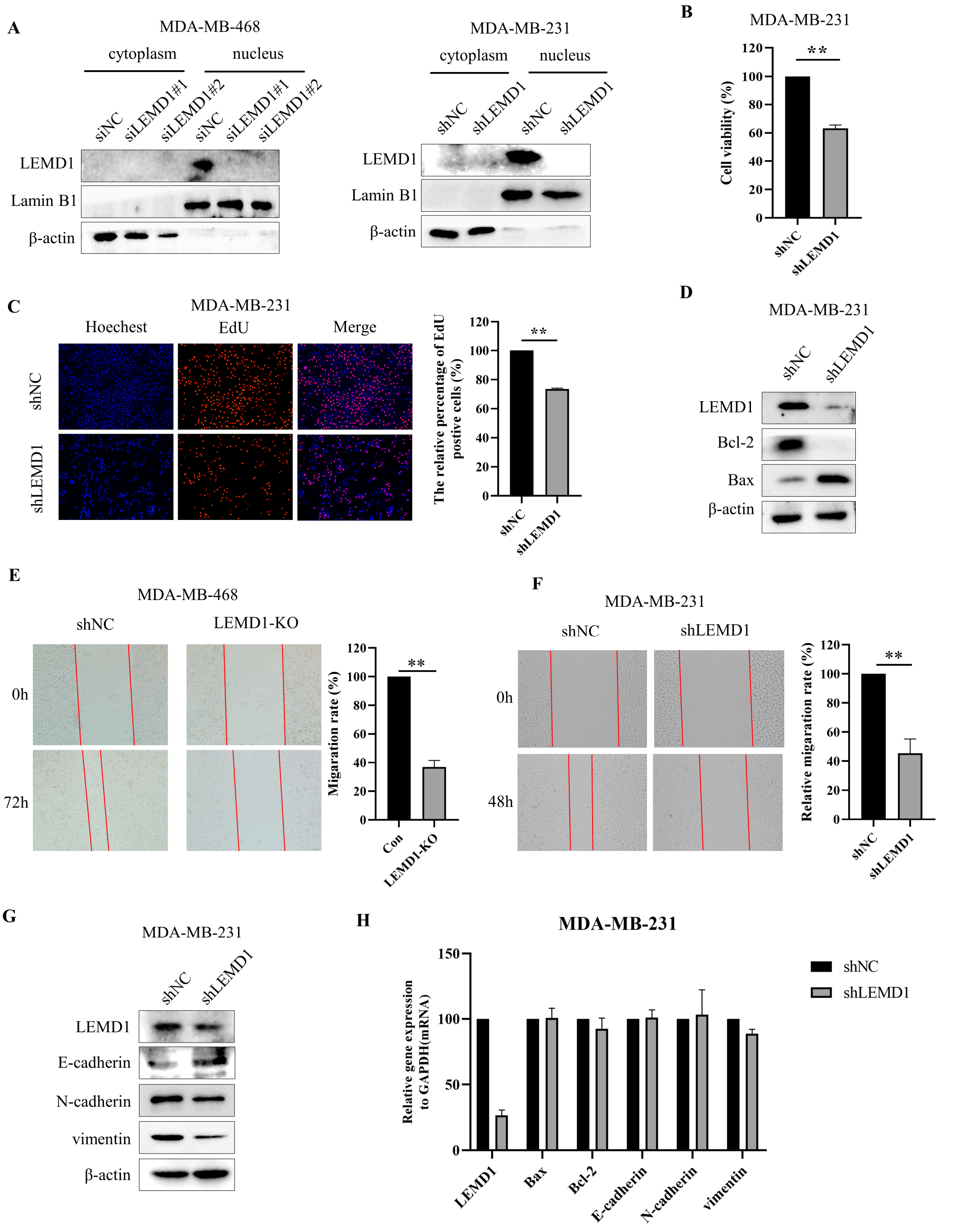

Figure S4. The localization and the oncogenic function of LEMD1 in TNBC cells. (A) The expression of LEMD1 was detected in cytoplasm and nucleus by Western blot. β-actin was used as the cytoplasmic loading control, and Lamin B1 was used as the nuclear loading control. **(B)** MDA-MB-231 cells were transfected with shRNA or a negative control, CCK-8 reagent was applied to examine cell viability. **p <0.01, n=3. **(C)** Cell proliferation of MDA-MB-231 cells was measured using EdU. Magnification, ×100. **p <0.01, n=3. **(D)** Western blot analysis of the expressions of Bcl-2 and Bax in MDA-MB-231 cells, β-actin was used as a loading control. **(E)** Wound healing assays of LEMD1-knockout MDA-MB-468 cells. Magnification, ×100. **p <0.01, n=3. **(F)** Wound healing assays of LEMD1-knockdown MDA-MB-231 cells. Magnification, ×100. **p <0.01, n=3. **(G)** Western blot

analysis of the expressions of E-cadherin, N-cadherin and vimentin in MDA-MB-231 cells, β-actin was used as a loading control. **(H)** qPCR analysis of the mRNA expressions of Bax, Bcl-2, E-cadherin, N-cadherin and vimentin in MDA-MB-231 cells.

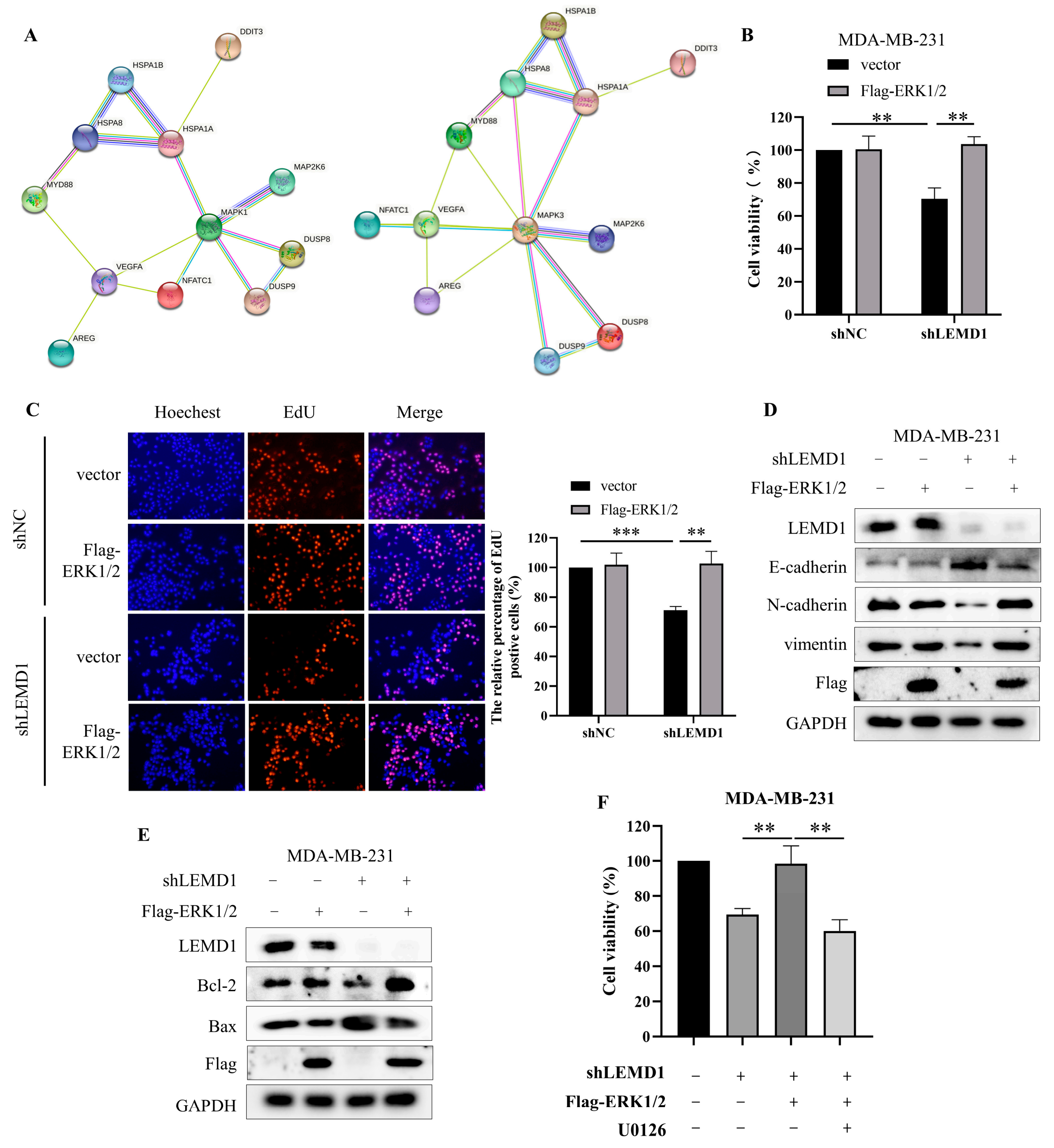

Figure S5. LEMD1 promotes the cell proliferation and invasion by activating ERK in TNBC. (A) The PPI network for ERK1 (MAPK3), ERK2 (MAPK1) and the 11 differential genes with significant variation in MAPK signaling pathway from RNA-seq. LEMD1-knockdown MDA-MB-231 cells were transfected with ERK plasmid or empty vector plasmid. **(B)** Cell viability was measured using the CCK8 assay. **(C)** Cell proliferation was measured by EdU assay. Magnification, ×200. **(D)** The protein expressions of EMT markers including E-cadherin, N-cadherin, vimentin, and **(E)** The expressions of Bcl-2 and Bax in LEMD1-knockdown MDA-MB-231 cells were measured by Western blot. GAPDH was used as a loading control. **(F)** LEMD1-knockdown MDA-MB-231 cells were transfected

with ERK plasmid and then treated with U0126 for 24h. Cell viability was measured using the CCK8 assay.

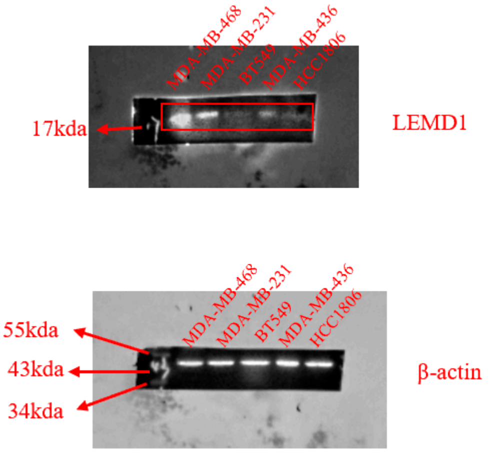

Figure S6A TNBC cell lines

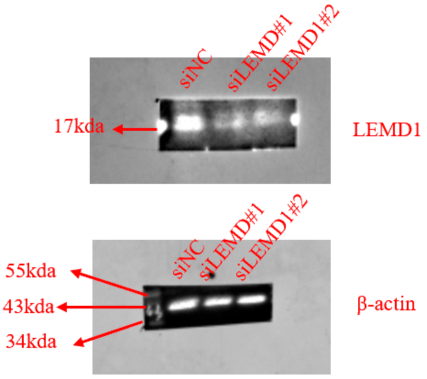

Figure S6A MDA-MB-468-knockdown

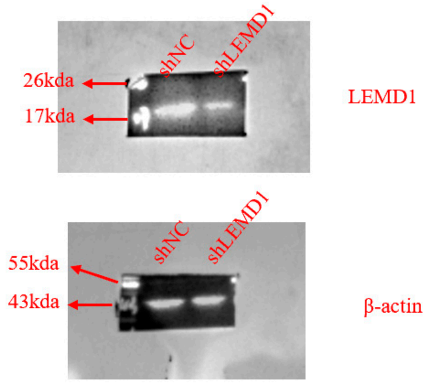

Figure S6A MDA-MB-231-knockdown

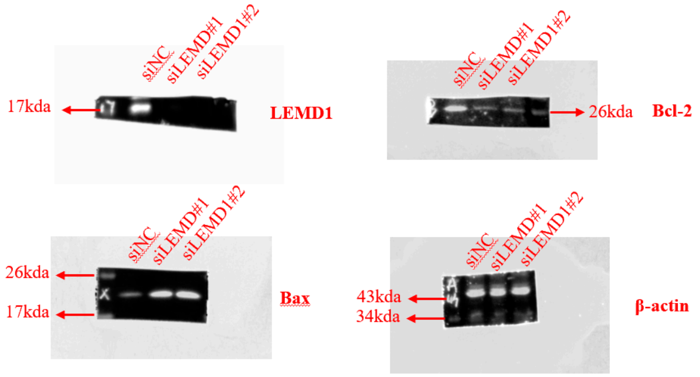

Figure S6A MDA-MB-468-apoptosis

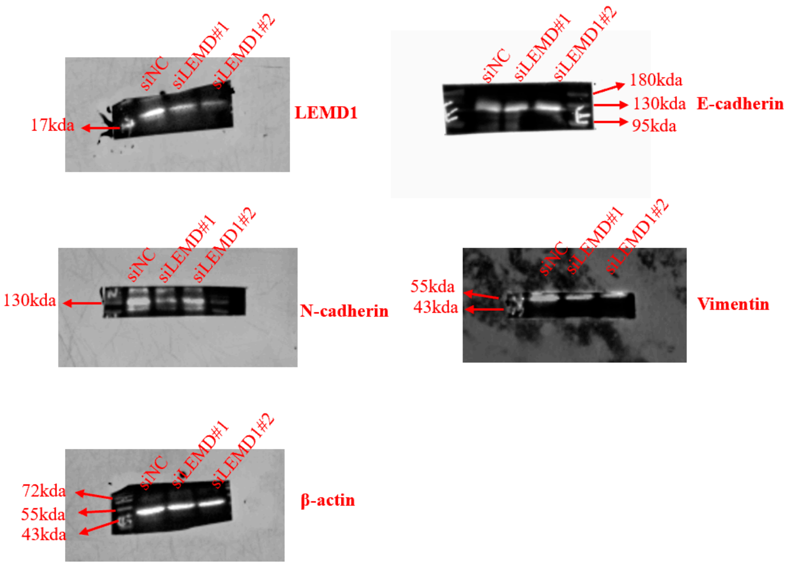

Figure S6A MDA-MB-468-EMT

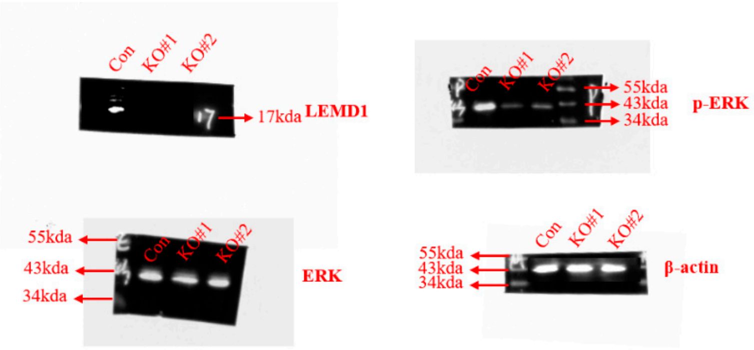

Figure S6B MDA-MB-468-ERK

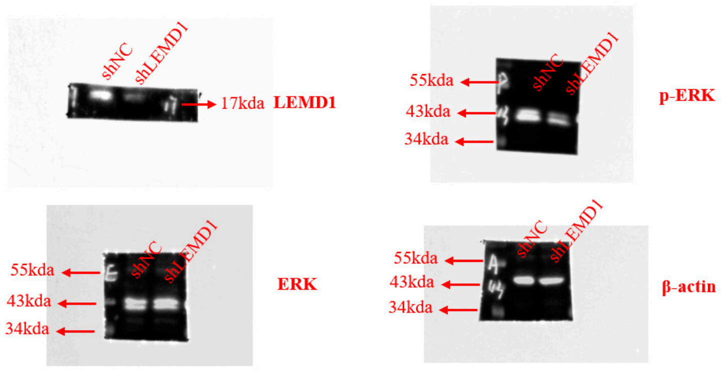

Figure S6B MDA-MB-231-ERK

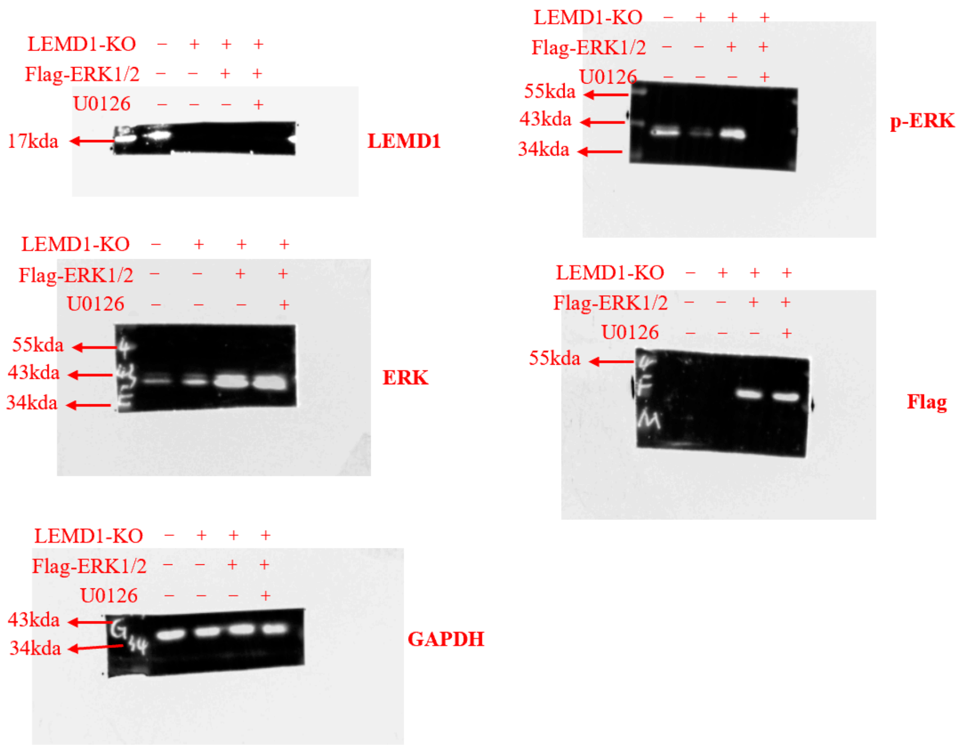

Figure S6B MDA-MB-468-U0126

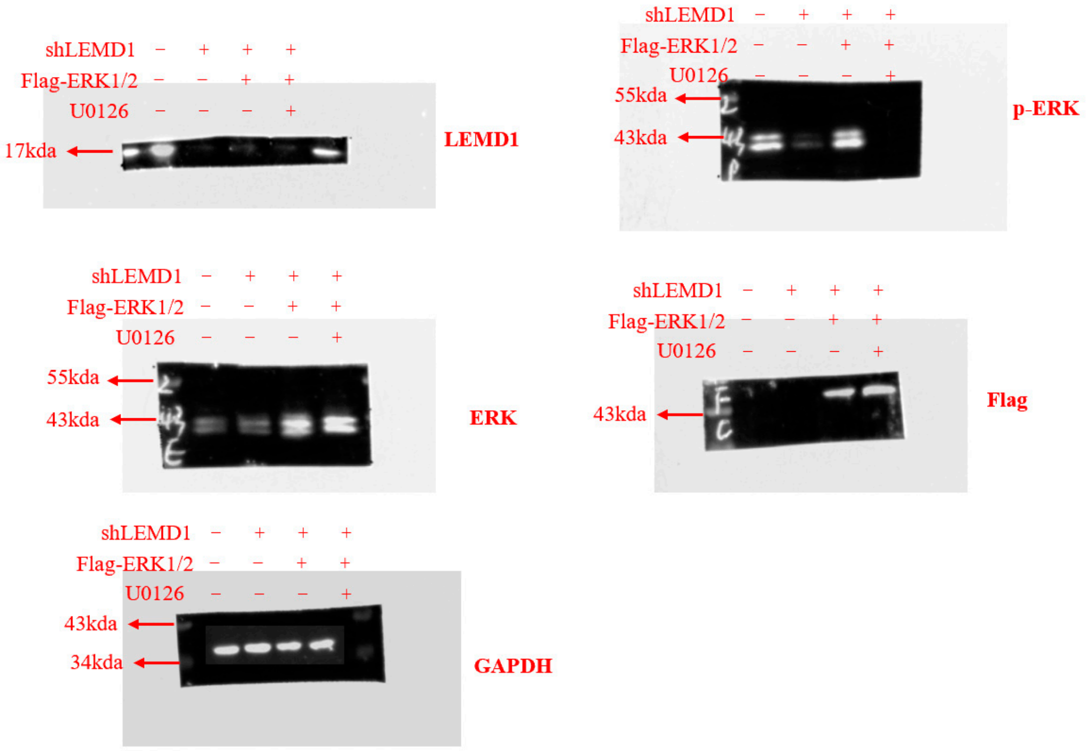

Figure S6B MDA-MB-231-U0126

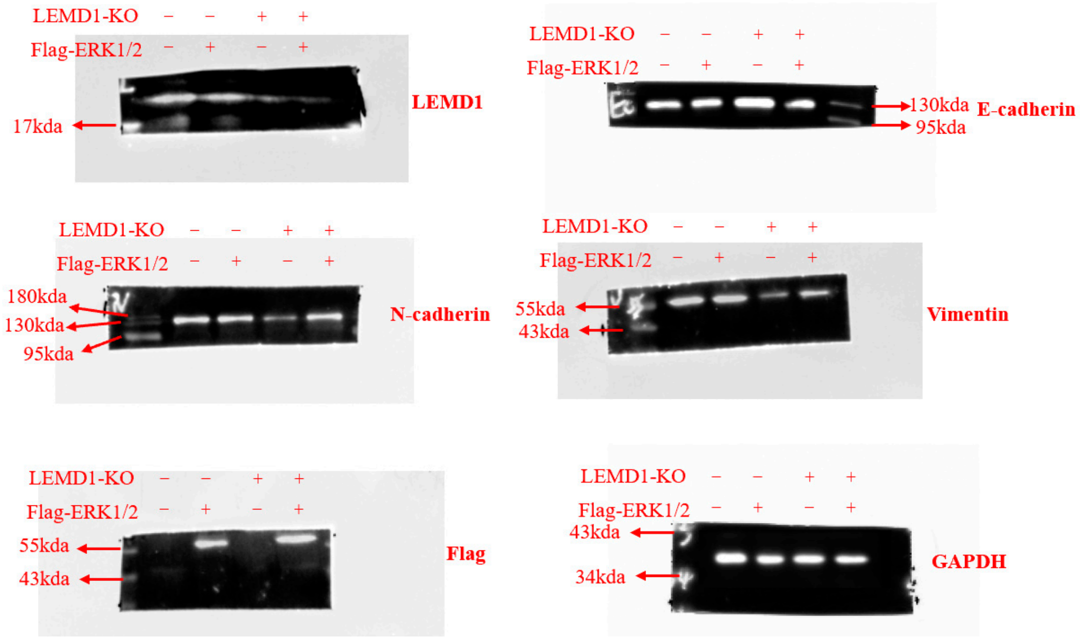

Figure S6B MDA-MB-468-EMT

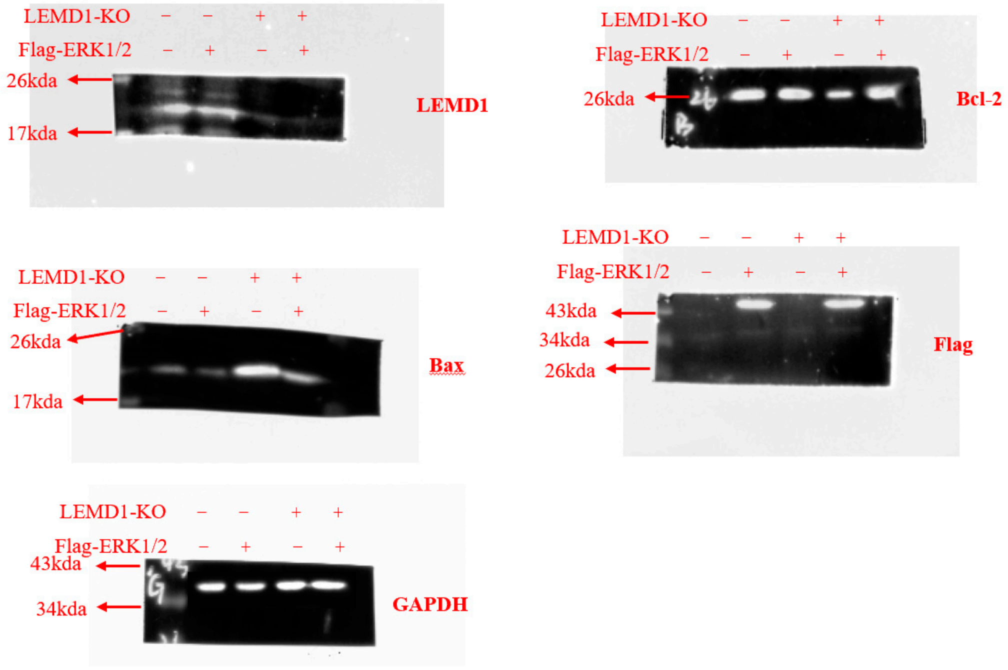

Figure S6B MDA-MB-468-apoptosis

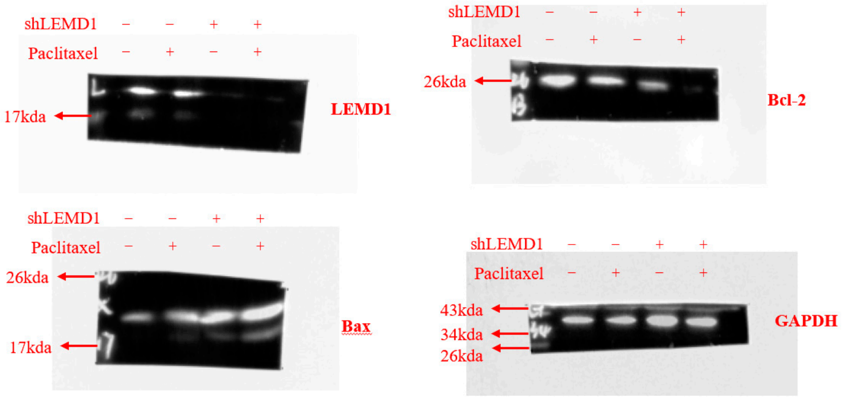

Figure S6C MDA-MB-231-apoptosis
